# Transoceanic Dispersal and Subsequent Diversification on Separate Continents Shaped Diversity of the *Xanthoparmelia pulla* Group (Ascomycota)

**DOI:** 10.1371/journal.pone.0039683

**Published:** 2012-06-20

**Authors:** Guillermo Amo de Paz, Paloma Cubas, Ana Crespo, John A. Elix, H. Thorsten Lumbsch

**Affiliations:** 1 Departamento de Biología Vegetal II, Facultad de Farmacia, Universidad Complutense de Madrid, Madrid, Spain; 2 Research School of Chemistry, Australian National University, Canberra, Australian Capital Territory, Australia; 3 Department of Botany, The Field Museum, Chicago, Illinois, United States of America; Nanjing Agricultural University, China

## Abstract

In traditional morphology-based concepts many species of lichenized fungi have world-wide distributions. Molecular data have revolutionized the species delimitation in lichens and have demonstrated that we underestimated the diversity of these organisms. The aim of this study is to explore the phylogeography and the evolutionary patterns of the *Xanthoparmelia pulla* group, a widespread group of one of largest genera of macrolichens. We used a dated phylogeny based on nuITS and nuLSU rDNA sequences and performed an ancestral range reconstruction to understand the processes and explain their current distribution, dating the divergence of the major lineages in the group. An inferred age of radiation of parmelioid lichens and the age of a *Parmelia* fossil were used as the calibration points for the phylogeny. The results show that many species of the *X. pulla* group as currently delimited are polyphyletic and five major lineages correlate with their geographical distribution and the biosynthetic pathways of secondary metabolites. South Africa is the area where the *X. pulla* group radiated during the Miocene times, and currently is the region with the highest genetic, morphological and chemical diversity. From this center of radiation the different lineages migrated by long-distance dispersal to others areas, where secondary radiations developed. The ancestral range reconstruction also detected that a secondary lineage migrated from Australia to South America via long-distance dispersal and subsequent continental radiation.

## Introduction

Methods for delimiting species, the fundamental taxonomic unit, have always fascinated evolutionary biologists [1]–[3]. Understanding the circumscription of species is important for biological and ecological studies and for conservation issues. However, the main challenge is to recognize species in organisms with relatively simple morphologies. In lichenized fungi traditional species circumscriptions are based on phenotypic characters, such as thallus and ascomatal morphology or chemical characters. However, there is a growing body of evidence from molecular studies that the traditional morphology-based species circumscriptions are insufficient to represent the diversity in lichenized ascomycetes [4]–[26]. A number of DNA sequence-based phylogenetic studies revealed the presence of distinct lineages within currently delimited species. Subsequent, detailed studies often revealed previously overlooked morphological subtleties or chemical differences among those clades and authors often refer to these as “semi-cryptic” species [8].

On a par with the phenotypic-based species circumscription, researchers often accepted wide distribution ranges for species occurring on different continents. This was at least partially due to a common belief by lichenologists in the “everything is everywhere” hypothesis [27], [28] applied to fungi, as discussed elsewhere [8], [29]. In several cases molecular data assisted in a better understanding of the biogeography of lichen-forming fungi where taxa were shown to represent different species on different continents, e.g. the *Leptogium furfuraceum* aggr. on different continents [18], *Melanelixia glabra* s. lat. in Europe and North America [14], *Parmelina quercina* s. lat. on different continents [4], *Physcia aipolia* aggr. in Europe and Australia [9], *Xanthoparmelia* spp. in North America and Australia [15], [16]. In the *Leptogium furfuraceum* aggr. complex sister-group relationships were found between populations from the same hemispheres which were incongruent with previous classifications based on morphological differences [18], and the dated phylogeny indicated that the species had migrated via transoceanic dispersal to different continents.

Here we report another case of a group of lichenized fungi where transoceanic dispersal to different continents is correlated with the phylogenetic lineages. The group studied here is the *Xanthoparmelia pulla* group which belongs to the family Parmeliaceae. This family represents one of the largest families of lichenized fungi [30], [31]. The main clade of the family is the parmelioid clade with almost 2000 species [32] currently classified in 27 genera, with *Xanthoparmelia* being the largest with over 800 accepted species [33]. The species in this genus characteristically occur on siliceous rocks or soil, predominantly in arid to subarid regions, with a center of distribution in the southern hemisphere. The genus is characterized by having cell wall polysaccharides of the *Xanthoparmelia*-type, small ascospores with an arachiform vacuolar body [34], and the presence of a pored epicortex [33], [35]. It has been hypothesized that the genus diversified in a rapid radiation following a shift towards drier habitats at the base of the *Xanthoparmelia* clade [36] leading to the high current diversity.

**Table 1:** Main differences of the species of *Xanthoparmelia pulla* group studied in this paper [38], [44], [47], [59], [60].

	Characters and distribution				
Species	Morphology of lobes	Isidia	Lower surface	Chemistry	Distribution
*X. atroviridis*	1–2 mm broad, subirregular, contiguous to imbricate	Absent	Black, moderately rhizinate, rhizines concolorous, up to 0.4 mm long.	Medulla: Hypoconstictic, hypostictic, hyposalazinic acids; Cortex: HNO_3_ + violet	South Africa
*X. caliginosa*	1–2.5 (−3.5) mm broad, subirregular, contiguous to imbricate	Sparse to crowded and areolate. Isidia erumpent, globose to cylindrical, 0.1–0.6 (−0.8) mm tall.	Dark brown to black, moderately rhizinate, rhizines more or less concolorous, up to 0.6 mm long.	Medulla: Olivetoric acid. Cortex: HNO_3_ + blue-green	South Africa
*X. delisei*	1–4 mm broad, sublinear to irregular, flat to slightly concave or convex, becoming laciniate, often imbricate and entangled	Absent	Dark brown to black, often paler near the apices, moderately to densely rhizinate, the rhizines simple and concolorous with the lower surface, to 1 mm long	Medulla: glomelliferic, glomellic, perlatolic acids; ± gyrophoric acid. Cortex: HNO_3_ + blue-green	Europe, Asia, Africa, Australia, Macaronesia, South America
*X. fissurina*	1–3 mm broad, contiguous to imbricate or entangled	Absent	Pale tan to pale brown, moderately rhizinate, rhizines concolorous, to 1 mm long	Medulla: hypostictic, hypoconstictic, hyposalazinic acids, unknown compounds. Cortex: HNO_3_ + blue-green	South Africa and South America
*X. glabrans*	0.5–3.0 mm broad, sublinear to linear-elongate, imbricate to loosely entangled.	Absent	black, dull, slightly rugulose, moderately rhizinate; rhizines black, simple or fasciculate, to 1 mm long	Medulla: alectoronic acid; ± a-collatolic and gyrophoric acids. Cortex: HNO_3_ + blue-green	Australia, Europe, Africa, South America, New Zealand
*X. imitatrix*	0.5–3.0 mm broad, sublinear to linear-elongate, imbricate to laciniate entangled, rarely developing subfruticose branches	Absent	Dark brown to black, sparsely to moderately rhizinate, rhizines simple, to 1.5 mm long	Medulla: physodic acid; ±4-O-methylphysodic and alectoronic acids. Cortex: HNO_3_ + blue-green	Australia, Africa, South America, New Zealand
*X. lineella*	0.1–0.5mm broad, linear and dichotomously branched and entangled	Absent	Black, sparsely rhizinate, rhizines concolorous, to 1 mm long	Medulla: physodic acid; Cortex: HNO_3_ + blue-green	South Africa
*X. loxodes*	(0.5-)1–3(−5) mm broad, subirregular to sublinear, contiguous to entangled.	Sparsely to densely isidiate, isidia more or less spherical and distinctly pustular, erumpent	Dark brown to black, smooth to somewhat rugulose, moderately rhizinate, rhizines concolorous, to 1 mm long	Medulla: glomelliferic, glomellic and perlatolic acids; ±gyrophoric acid. Cortex: HNO_3_ + blue-green	Europe, North Africa, Asia, North America, Macaronesia
*X. luteonotata*	(0.5-)1–3 mm broad, sublinear to irregular, discrete to imbricate, rarely developing subfruticose branches	Absent	Pale tan to pale brown, moderately to densely rhizinate, rhizines simple, to 0.5 mm long	Medulla: ± divaricatic and stenosporic acids; ± gyrophoric acid. Cortex: HNO_3_ + blue-green	Australia, Europe, Africa, New Zealand
*X. pokornyi*	1–2 mm broad, sublinear to linear, discrete to loosely imbricate or entangled	Absent	Pale tan to brown, moderately to sparsely rhizinate, rhizines concolorous or darkening, to 1 (−1.5) mm long	Medulla: stenosporic acid; ± gyrophoric and divaricatic acids. Cortex: HNO_3_ + blue-green	Europe, Asia
*X. perrugata*	1–3 (−5) mm broad, sublinear to linear-elongate, discrete to imbricate or entangled.	Absent	Dark brown to black, moderately to densely rhizinate, rhizines simple, to 1.5 mm long.	Medulla: divaricatic acid; ± stenosporic, oxostenosporic, gyrophoric, lecanoric acids. Cortex: HNO_3_ + blue-green	Europe, North Africa, Australia, Asia
*X. pseudoglabrans*	1–2.5 mm broad, subirregular to sublinear, imbricate to entangled	Absent	Black; moderately rhizinate or rhizines rather parse, concolorous with the lower surface	Medulla: alectoronic acid; ± a-collatolic acid. Cortex: HNO_3_ -	South Africa
*X. pulla*	1–3 (−5) mm broad, sublinear to linear-elongate, discrete to imbricate or entangled	Absent	Dark brown to black, moderately to densely rhizinate, rhizines simple, to 1.5 mm long	Medulla: stenosporic acid; ± divaricatic, gyrophoric, perlatolic, 4-O-demethylstenosporic acid, oxostenosporic acids. Cortex: HNO_3_ + blue-green	Europe, Australia, New Zealand, Africa
*X. pulloides*	1–2 mm broad, subirregular to sublinear, contiguous to subimbricate	Absent	Black, moderately rhizinate, rhizines concolorous, to 0.5 mm long	Medulla: constipatic and protoconstipatic acids; ± gyrophoric acid. Cortex: HNO_3_ + blue-green	Macaronesia, Asia
*X. quintarioides*	1–2.5 (−3) mm broad, strongly convex and short-flabellate, discrete but close to more or less contiguous	Absent	Tan to pale brown, sparsely to moderately rhizinate, the rhizines short and hapterate	Medulla: hypostictic, hypoconstictic, cryptostictic acids; ± hyposalazinic acid. Cortex: HNO_3_ + blue-green	South Africa
*X. ryssolea*	1–3 mm broad, linear elongate, subterete, convex.	Absent	Pale yellow-brown to red-brown, canaliculate, sparsely rhizinate, rhizines concolorous, to 0.6 mm long.	Medulla: stenosporic acid; ± gyrophoric, oxostenosporic, divaricatic acids. HNO_3_ + blue-green	Europe, Asia
*X. squamans*	1–2 mm broad, sublinear, imbricate to contiguous	Absent	Dark brown to black, moderately to sparsely rhizinate, rhizines concolorous, to 1 mm long	Medulla: hypostictic, hypoconstictic, hyposalazinic acids. HNO_3_ + blue-green	South Africa, South America, New Zealand
*X. subhosseana*	1–2 mm broad, subirregular, contiguous to slightly imbricate	Sparsely to densely isidiate. Isidia pustular, erumpent	Dark brown to black, moderately rhizinate, rhizines concolorous, to 0.6 mm long	Medulla: hypostictic, hyposalazinic, hypoconstictic acids. Cortex: HNO_3_ + blue-green	South Africa, North America, South America, New Zealand
*X. subimitatrix*	0.5–2.0 mm broad, sublinear to subirregular, discrete to subimbricate	Absent	Pale tan to brown, moderately rhizinate, rhizines simple, brown or often blackened, to 0.8 mm long	Medulla: physodic acid and alectoronic acids. Cortex: HNO_3_ + blue-green	South Africa, Australia.
*X. subincerta*	0.5–1 mm broad, flat, sublinear, more or less imbricate	Isidia cylindrical, simple or densely branched, 0.08–0.5 mm tall. Apices syncorticate	Black, moderately rhizinate, rhizines simple, black, to 0.3 mm long	Medulla: glomelliferonic acid; ± loxodellonic and glomellonic acids. Cortex: HNO_3_ + blue-green	Australia, South Africa
*X. subprolixa*	1–3 (−5) mm broad, sublinear to linear-elongate, discrete to imbricate or entangled.	Absent	Dark brown to black, often paler at apices, moderately to densely rhizinate, rhizines simple, to 1.5 mm long	Medulla: divaricatic acid; ± stenosporic, nordivaricatic acids. Cortex: HNO_3_ + blue-green	Australia, New Zealand
*X. torulosa*	1.0–3.5 mm broad, sublinear to sublirregular, imbricate; laciniae at periphery and within thallus, ± subfruticose, sublinear to elongate, 0.3–1.0 mm broad.	Absent	Black, moderately to densely rhizinate; rhizines simple or occasionally tufted, slender.	Medulla: divaricatic acid; ± nordivaricatic, stenosporic acids. Cortex: HNO_3_ + blue-green	Australia
*X. verisidiosa*	1–3 mm broad, irregular to sublinear, flat, short and rounded, contiguous to imbricate	Sparsely to densely isidiate. Isidia cylindrical, simple or becoming densely branched, 0.2–1 mm tall. Apices syncorticate	Black, moderately to sparsely rhizinate, rhizines simple, black to 0.4 mm long	Medulla: alectoronic and a-collatolic acids. Cortex: HNO_3_ + blue-green	Australia, New Zealand, South Africa
*X. verrucella*	0.5–2 mm wide, irregular to sublinear, flat, imbricate to entangled.	Moderate to densely isidiate. Isidia cylindrical, simple or becoming branched, to 1 mm tall. Apices syncorticate	Black, sparsely to moderately rhizinate, rhizines simple, simple, black, to 0.4 mm long.	Medulla: divaricatic acid; ± stenosporic acid. Cortex: HNO_3_ + blue-green	Australia, New Zealand, South Africa
*X. verruculifera*	1–2 mm broad, subirregular to sublinear, contiguous to imbricate	Sparcely to densely isidiate. Isidia pustular, erumpent.	Dark brown to black, moderately rhizinate, rhizines concolorous, to 0.8 mm long	Medulla: divaricatic acid; ± stenosporic and gyrophoric acids. Cortex: HNO_3_ + blue-green	North Africa, Europe, North America

Previously, the *Xanthoparmelia pulla* group has been classified within the separate genus *Neofuscelia* based on the different cortical chemistry (having melanoid pigments and lacking usnic acid or atranorin, characteristic of the majority of *Xanthoparmelia* species) [37], [38]. A subsequent molecular study showed that the genus *Neofuscelia* was polyphyletic, with its clades scattered within *Xanthoparmelia* [33]. Consequently, the genus *Neofuscelia* was reduced to synonymy with *Xanthoparmelia,* as have other genera previously distinguished by cortical chemistry or growth form [33], [39]–[43]. The *Xanthoparmelia pulla* group is a monophyletic clade within the complete *Xanthoparmelia* clade, that includes the former Esslinger's *Xanthoparmelia pulla* species and other related species [44].

**Figure 1:**
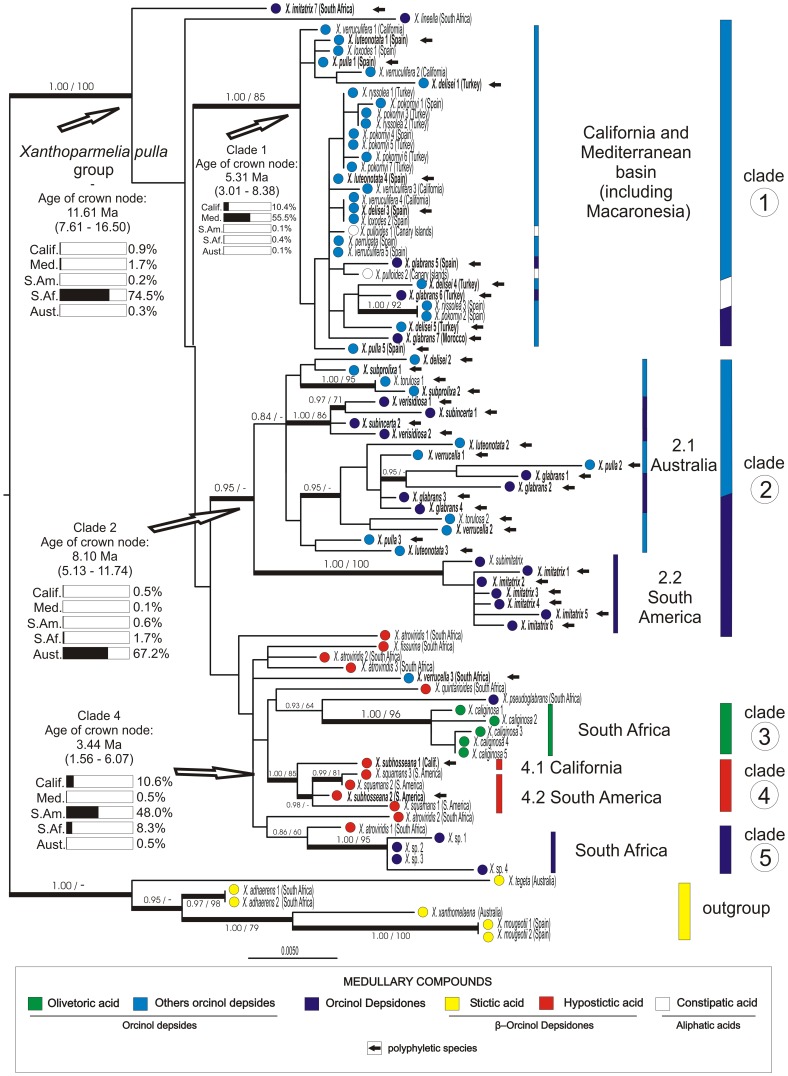
Phylogenetic relationships of the *Xanthoparmelia pulla* group based on nuITS and nuLSU rDNA sequences. Topology based on maximum-likelihood (ML) analyses. Posterior probabilities and bootstrap values are indicated on each branch. Branches with posterior probabilities under Bayesian analysis equal or above 0.95 and/or bootstrap values equal or above 70% under MP are in bold. Medullary compounds, and results of the divergence time estimation and ancestral range reconstruction analyses are shown. Black arrow head indicates the polyphyletic species.

**Figure 2:**
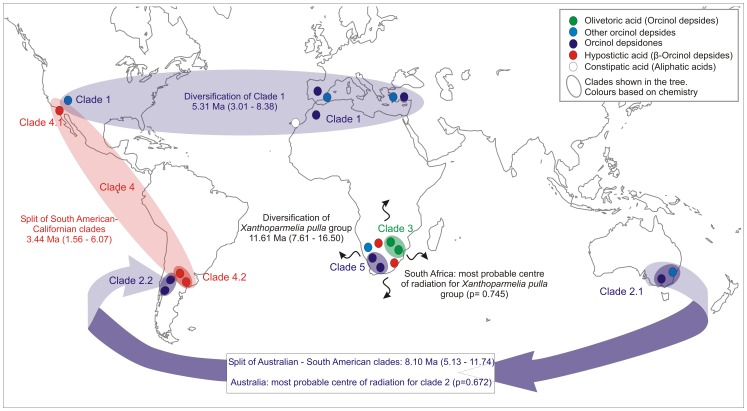
Schematic map showing the relationships between phylogeny, medullary compounds, ancestral range and divergence times estimation for the *Xanthoparmelia pulla* group.

Although the clades were largely incongruent with the current species circumscription, we found a correlation of the main clades of *X. pulla* group with their geographical distribution and chemical profile (Fig. 1, 2). Clade 1 includes specimens from California, Macaronesia and areas around the Mediterranean basin, all of which contain depsides and depsidones derived from the orcinol pathway or with aliphatic acids; clade 2 includes specimens from Australia (subclade 2.1) and South America (subclade 2.2) with depsides and depsidones derived from the orcinol pathway; clade 3 derives from South African specimens containing olivetoric acid; clade 4 specimens with hypostictic acid from California (subclade 4.1) and South America (subclade 4.2); and clade 5 specimens with physodic acid (orcinol depsidones) from South Africa.

The species delimitation within the *Xanthoparmelia pulla* group is currently based on a combination of morphological and chemical characters (Table 1). The morphological characters include the color of the lower surface, shape of the lobes, attachment to the substrate, and presence of vegetative propagules while the chemical differences pertain to upper cortical and medullary secondary metabolites. A number of the currently accepted species have a wide distribution spanning several continents. To address the species delimitation in this group and to test the hypothesis of widely distributed species we have generated a data set using two loci (nuITS rDNA, nuLSU rDNA) from specimens collected on different continents. The molecular data were used to perform phylogenetic reconstructions in a maximum likelihood (ML) and Bayesian (B/MCMC) framework. We have also estimated the timing of the diversification events leading to the main clades found in our study to discriminate between vicariance and long-distance dispersal as possible explanations for the current distribution patterns. A Bayesian-based approach of ancestral range reconstruction was used to identify potential areas in which the group and major clades within the group originated.

**Table 2:** Specimens used in this study with country of collection, voucher information and GenBank accession numbers.

			GenBank accession no.	
Species	Country	Herbarium acc. no.	nuITS	nuLSU
*Xanthoparmelia adhaerens* 1	South Africa	MAF-Lich 16212	HM125744	HM125766
*X. adhaerens* 2	South Africa	MAF-Lich 16213	HM125746	HM125768
*X. atroviridis* 1	South Africa	MAF-Lich 17163	**JQ912329**	**JQ912425**
*X. atroviridis* 1	South Africa	MAF-Lich 17168	**JQ912351**	**JQ912448**
*X. atroviridis* 2	South Africa	MAF-Lich 17158	**JQ912314**	**JQ912415**
*X. atroviridis* 2	South Africa	MAF-Lich 17154	**JQ912320**	**JQ912419**
*X. atroviridis* 3	South Africa	MAF-Lich 17153	**JQ912349**	**JQ912446**
*X. caliginosa* 1	South Africa	MAF-Lich 17157	**JQ912350**	**JQ912447**
*X. caliginosa* 2	South Africa	MAF-Lich 17150	**JQ912315**	**-**
*X. caliginosa* 3	South Africa	MAF-Lich 17156	**JQ912333**	**JQ912430**
*X. caliginosa* 4	South Africa	MAF-Lich 17152	**JQ912317**	**-**
*X. caliginosa* 5	South Africa	MAF-Lich 17186	**JQ912348**	**JQ912445**
*X. delisei* 1	Turkey	MAF-Lich 17139	**JQ912307**	**JQ912408**
*X. delisei* 2	Australia	MAF-Lich 7432	AY581067	AY578930
*X. delisei* 3	Spain	MAF-Lich 7659	AY581068	AY578931
*X. delisei* 4	Turkey	MAF-Lich 17134	**JQ912308**	**JQ912409**
*X. delisei* 5	Turkey	MAF-Lich 17135	**JQ912305**	**JQ912406**
*X. fissurina*	South Africa	MAF-Lich 17162	**JQ912353**	**JQ912450**
*X. glabrans* 1	Australia	CANB 746334	**JQ912291**	**JQ912393**
*X. glabrans* 2	Australia	CANB 746340	**JQ912289**	**JQ912391**
*X. glabrans* 3	Australia	MAF-Lich 7665	AY581069	AY578932
*X. glabrans* 4	Australia	CANB 681875.1	**JQ912290**	**JQ912392**
*X. glabrans* 5	Spain	MAF-Lich 9912	AY581072	AY578935
*X. glabrans* 6	Turkey	MAF-Lich 17137	**JQ912306**	**JQ912407**
*X. glabrans* 7	Morocco	MAF-Lich 17144	**JQ912286**	**JQ912388**
*X. imitatrix* 1	Chile	MAF-Lich 17132	**JQ912344**	**JQ912441**
*X. imitatrix* 2	Chile	MAF-Lich 17126	**JQ912288**	**JQ912390**
*X. imitatrix* 3	Chile	MAF-Lich 17123	**JQ912287**	**JQ912389**
*X. imitatrix* 4	Chile	MAF-Lich 17127	**JQ912342**	**JQ912439**
*X. imitatrix* 5	Chile	MAF-Lich 17122	**JQ912285**	**JQ912387**
*X. imitatrix* 6	Chile	MAF-Lich 17124	**JQ912326**	**JQ912422**
*X. imitatrix* 7	South Africa	MAF-Lich 17155	**JQ912352**	**JQ912449**
*X. lineella*	South Africa	MAF-Lich 17160	**JQ912319**	**JQ912418**
*X. loxodes* 1	Spain	MAF-Lich 7072	AY581076	AY578940
*X. loxodes* 2	Spain	MAF-Lich 6206	AY581070	AY578933
*X. luteonotata* 1	Spain	MAF-Lich 17120	**JQ912341**	**JQ912438**
*X. luteonotata* 2	Australia	CANB 746358	**JQ912293**	**-**
*X. luteonotata* 3	Australia	CANB 746366.1	**JQ912292**	**JQ912394**
*X. luteonotata* 4	Spain	MAF-Lich 17119	**JQ912340**	**JQ912437**
*X. mougeotii* 1	Spain	MAF-Lich 6802	AY37006	AY578966
*X. mougeotii* 2	Spain	MAF-Lich 9916	AY581100	AY578967
*X. perrugata*	Spain	MAF-Lich 17118	**JQ912324**	**-**
*X. pokornyi* 1	Spain	MAF-Lich 6052	AY037005	AY578934
*X. pokornyi* 2	Spain	MAF-Lich 9908	AY581075	AY578939
*X. pokornyi* 3	Turkey	MAF-Lich 17140	**JQ912310**	**JQ912411**
*X. pokornyi* 4	Spain	MAF-Lich 17117	**JQ912323**	**-**
*X. pokornyi* 5	Turkey	MAF-Lich 17143	**JQ912313**	**JQ912414**
*X. pokornyi* 6	Turkey	MAF-Lich 17142	**JQ912312**	**JQ912413**
*X. pokornyi* 7	Turkey	MAF-Lich 17136	**JQ912304**	**JQ912405**
*X. pseudoglabrans*	South Africa	MAF-Lich 17161	**JQ912316**	**JQ912416**
*X. pulla* 1	Spain	MAF-Lich 17115	**-**	**JQ912420**
*X. pulla* 2	Australia	CANB 739130.1	**JQ912294**	**JQ912395**
*X. pulla* 3	Australia	CBG 9810185	**JQ912295**	**JQ912396**
*X. pulla* 5	Spain	MAF-Lich 6794	AY581071	AJ 421433
*X. pulloides* 1	Spain	MAF-Lich 17121	**JQ912347**	**JQ912444**
*X. pulloides* 2	Spain	MAF-Lich 6784	AY037004	AY578936
*X. quintarioides*	South Africa	MAF-Lich 17159	**JQ912318**	**JQ912417**
*X. ryssolea* 1	Turkey	MAF-Lich 17141	**JQ912311**	**JQ912412**
*X. ryssolea* 2	Turkey	MAF-Lich 17138	**JQ912309**	**JQ912410**
*X. ryssolea* 3	Spain	MAF-Lich 17116	**JQ912322**	**-**
*X. sp.* 1	South Africa	MAF-Lich 17166	**JQ912330**	**JQ912426**
*X. sp.* 2	South Africa	MAF-Lich 17167	**JQ912331**	**JQ912427**
*X. sp.* 3	South Africa	MAF-Lich 17165	**-**	**JQ912429**
*X. sp.* 4	South Africa	MAF-Lich 17164	**JQ912339**	**JQ912436**
*X. squamans* 1	Chile	MAF-Lich 17128	**JQ912325**	**JQ912421**
*X. squamans* 2	Chile	MAF-Lich 17129	**JQ912327**	**JQ912423**
*X. squamans* 3	Chile	MAF-Lich 17131	**JQ912343**	**JQ912440**
*X. subhosseana* 1	USA	MAF-Lich 17149	**JQ912337**	**JQ912434**
*X. subhosseana* 2	Chile	MAF-Lich 17133	**JQ912345**	**JQ912442**
*X. subimitatrix*	Chile	MAF-Lich 17130	**JQ912328**	**JQ912424**
*X. subincerta* 1	Australia	CANB 746346.1	**JQ912296**	**JQ912397**
*X. subincerta* 2	Australia	MAF-Lich 7494	AY581073	AY578937
*X. subprolixa* 1	Australia	MAF-Lich 7667	AY581074	AY578938
*X. subprolixa* 2	Australia	CANB 746355	**JQ912297**	**JQ912398**
*X. tegeta*	Australia	MAF-Lich 7523	AY581107	AY578975
*X. torulosa* 1	Australia	CANB 746363.1	**JQ912299**	**JQ912400**
*X. torulosa* 2	Australia	CANB 746351	**JQ912298**	**JQ912399**
*X. verisidiosa* 1	Australia	CANB 746341.1	**JQ912301**	**JQ912402**
*X. verisidiosa* 2	Australia	CANB 746345.1	**JQ912300**	**JQ912401**
*X. verrucella* 1	Australia	CANB 746353	**JQ912303**	**JQ912404**
*X. verrucella* 2	Australia	CANB 746349	**JQ912302**	**JQ912403**
*X. verrucella* 3	South Africa	MAF-Lich 17151	**JQ912332**	**JQ912428**
*X. verruculifera* 1	USA	MAF-Lich 17146	**JQ912334**	**JQ912431**
*X. verruculifera* 2	USA	MAF-Lich 17147	**JQ912336**	**JQ912433**
*X. verruculifera* 3	USA	MAF-Lich 17145	**JQ912338**	**JQ912435**
*X. verruculifera* 4	USA	MAF-Lich 17148	**JQ912335**	**JQ912432**
*X. verruculifera* 5	Spain	MAF-Lich 17114	**JQ912321**	**-**
*X. xanthomelaena*	Australia	MAF-Lich 16447	HM125740	HM125761

New sequences are in bold.

## Results

### Phylogenetic analyses

One hundred sixty-eight DNA sequences of ITS and nuLSU rDNA of 88 representative specimens of *Xanthoparmelia* were assembled. One hundred forty of these sequences were newly generated in this study. The specimens included 25 currently accepted species in the *Xanthoparmelia pulla* group, four unassigned specimens, and six samples of four species as outgroup. A data matrix of 1283 unambiguously aligned characters, with 454 characters in the ITS and 829 characters in the nuLSU rDNA was used for phylogenetic analyses. The data set included 1081 constant characters. The general time-reversible model with a gamma distribution and invariant model of rate heterogeneity (GTR+I+G) was employed for analyses of the single-loci and concatenated data sets. Since no strongly supported conflicts between the two single-locus ML phylogenetic trees were detected, a combined data set was analyzed. In the B/MCMC analysis of the combined data set, the likelihood parameters in the sample had the following averaged values for the partitioned data set (± standard deviation): base frequencies π(A)  = 0.25 (±1.54E-4), π(C)  = 0.24 (±1.42E-4), π(G)  = 0.28 (±1.58E-4), π(T)  = 0.23 (±1.51E-4); rate matrix r(AC)  = 4.42 (±1.43E-4), r(AG)  = 0.23 (±8.35E-4), r(AT)  = 9.53 (±2.19E-4), r(CG)  = 4.82 (±1.45E-4), r(CT)  = 0.54 (±9.26E-4), r(GT)  = 2.98 (±1.11E-4) and the gamma shape parameter α  = 0.21 (±3.86E-4). The likelihood parameters in the sample had a mean likelihood of LnL  = −4608.25 (±0.49), while the ML tree had a likelihood of LnL = −4163.64.

The phylogenetic estimates of the ML and B/MCMC analyses were congruent, hence only the ML tree (Fig. 1) is shown here. Specimens of the *Xanthoparmelia pulla* group form a strongly supported monophyletic group with five main, mostly well-supported, clades (Fig. 1). The clades do not agree with the current species circumscription, with 11 species being polyphyletic, five of them with specimens from different continents entering different clades. For example, all the Northern Hemisphere specimens identified as *X. luteonotata*, *X. pulla*, *X. delisei* or *X. glabrans* belong to clade 1 while all the Australian specimens of the same species belong to clade 2.1. Similarly, specimens of *X. imitatrix* from South America belong to clade 2.2 and are not directly related to the South African specimen.

### Estimates of divergence times and ancestral range reconstructions

A Bayesian phylogenetic tree was dated to estimate the age of the *X. pulla* group and its main clades. The results of the divergence time analysis are summarized in Fig. 2, and the whole parmelioid tree is shown as supp. mat. (Fig. S1). The *Xanthoparmelia pulla* group started to diversify around 11.61 Ma (7.61 – 16.50 Ma), the age of the crown node of clade 1 was estimated at 5.31 Ma (3.01 – 8.38 Ma), the ancestor of clade 2 around 8.10 Ma (5.13 – 11.74 Ma), and the crown of clade 4 around 3.44 Ma (1.56 – 6.07 Ma).

The results of the ancestral range reconstruction analyses are summarized in Figs. 1 and 2. This established that South Africa was the most likely origin of the *X. pulla* group, with a marginal probability of 0.745, indicating localized uncertainty. The four other areas explored (South America, Australia, California and the Mediterranean basin) were rejected with probabilities below 0.05. For the base of clade 1, the Mediterranean basin was reconstructed as the most likely ancestral range with a marginal probability of 0.555, but California could not be rejected (probability of 0.104). For clade 2, Australia was recovered as the most likely ancestral area with marginal probability of 0.672; while South America, the other area from which specimens of this clade occur, was rejected as potential ancestral area (p<0.05); similarly, California, the Mediterranean basin and South Africa were also rejected as ancestral areas. South America was found to be the most likely origin for clade 4 (which also includes specimens from California and South America) with a marginal probability of 0.48, although neither California nor South Africa were rejected (probabilities of 0.106 and 0.083, respectively). Australia and the Mediterranean basin were rejected as ancestral ranges for clade 4.

## Discussion

Understanding the diversity and delimiting species in lichenized fungi has been a long standing challenge and current studies using molecular data have dramatically changed our ability to distinguish species in this group [8], [23], [45]. The *Xanthoparmelia pulla* group is a good example for illustrating the difficulties in in distinguishing species by morphology due to the remarkable plasticity of morphological characters in this group. Consequently, secondary metabolites have played an important role in delimiting species in this group [44], [46], [47]. Following the current classification using a combination of vegetative morphology and secondary chemistry, a number of species have broad geographical distributions spanning several continents.

Here we have used molecular data to investigate the current classification within the group and attempt to explain their distribution. We used likelihood-based and Bayesian approaches to investigate the evolutionary origin of the group and timing of speciation events. Hopefully such data will reveal evolutionary patterns so we may develop a framework for their taxonomic classification which better reflects the phylogenetic relationships in the *X. pulla* group. Our results clearly indicate that the species as currently delimited are polyphyletic (Fig. 1). This is consistent with results from other studies of *Xanthoparmelia* species believed to occur on different continents which were subsequently found to represent distinct lineages [15], [16]. Further, similar patterns have been found in other groups of lichenized fungi [4], [14], [18], [48].

The ancestral range reconstruction points to South Africa as the most likely origin of the *X. pulla* group. Although there is a certain degree of uncertainty in the reconstruction (marginal probability of 0.745), the analysis rejected other areas as potential ancestral areas for the group. Interestingly, South Africa has the highest morphological and chemical diversity within the group and the specimens studied here belong to different, unrelated lineages (Fig. 1). South African specimens containing olivetoric acid cluster in clade 3 and those with physodic acid in clade 5. The phylogenetic relationships of other specimens from South Africa with hypostictic acid, physodic acid or other orcinol depsides and depsidones are still unresolved. The South African specimens show remarkable morphological variability, including subcrustose and foliose species. Further, many *Xanthoparmelia* species occur in arid climates and the diversification of *X. pulla* group occurred around 11.61 Ma (7.61 – 16.50). At this time the Cape Region underwent a major aridification [49], which may be responsible for the rapid radiation and current richness of the Cape flora. Thus, it is likely that the *X. pulla* group originated in South Africa around the same time. Unlike most Cape region elements in flowering plants, the species of the *X. pulla* group subsequently extended their distribution by transoceanic dispersal.

Within the *X. pulla* group, the five lineages identified are characterized by the presence of different substance classes and in some cases they diverged secondarily in different geographical areas. The correlation between chemical pathways and the lineages found in molecular studies has also been found in Pertusariaceae among lichenized fungi [50]–[52].

Clade 1 includes specimens containing orcinol depsides and depsidones that occur in California and the Mediterranean basin. Neither internally supported subclades nor a geographical pattern was found within this clade, and specimens with different phenotypical characters from different geographical areas are intermingled. In fact, shifting between orcinol depsides and depsidones can occur by one-step transformations [46]. By contrast, in other genera (e.g. *Melanelixia, Parmelina, Leptogium*) with similar disjunct distributions (North America and the Mediterranean basin), the geographical distribution correlates with the clades found in the molecular study. In the *X. pulla* group this pattern was not found, possibly due to insufficient sampling or absence of a phylogenetic signal in the markers used. This might be due to the slower evolutionary rates of lichenized fungi from arid and subarid regions compared to oceanic parmelioid lichens [36]. The diversification age of clade 1 was estimated at 5.31 Ma (3.01 – 8.38 Ma), at the end of the Miocene, which was a geological period when numerous groups radiated in arid conditions. The Mediterranean region was suggested as the ancestral area of this clade by the ancestral range analysis, but the result was poorly supported.

Clade 2 also contains species with orcinol depsides and depsidones and comprises two disjunct lineages, one occurring in Australia (clade 2.1) and the other in South America (2.2). The estimated age for clade 2 (8.10 Ma; 5.13 – 11.74 Ma) rules out the possibility of vicariance, since the breakup of Australia, Antarctica and South America occurred between 35–52 Ma ago [53]. The ancestral range reconstruction points to Australia as the ancestral area of clade 2. This would be consistent with long distance dispersal from Australia to South America, a phenomenon frequently found in many plants groups [54]. Within the Australian clade several strongly supported lineages are not consistent with the current species delimitation of the group, indicating that the phenotypical characters used to distinguish species in the group have limited phylogenetic validity. Similar disparities between phylogenetic relationships and current species delimitations were found within the yellow *Xanthoparmelia* species from western North American [55], [56].

Clade 4 includes specimens containing hypostictic acid from California and South America. Here again all the South American specimens form a monophyletic group. Specimens of *X. subhosseana* occurring in different continents are not closely related. The most likely ancestral origin of clade 4 is South America (marginal probability of 0.48), although neither California nor South Africa could be rejected.

Our study indicates that the *X. pulla* group started to radiate during the Miocene in South Africa, where the highest diversity of this group is found. From this region, different lineages with distinct secondary metabolites belonging to different chemical pathways were dispersed to other regions, where they experienced rapid and more recent radiations. In some cases our results showed that the sympatric species of the *X. pulla* group in an area belong to distantly related groups. For example, the Californian *X. pulla* flora includes species from clade 1 and clade 4.1, the latter most probably having migrated from South America. Indeed our study indicated that the current taxonomic circumscription of species in the group does not agree with the evolutionary hypotheses inferred by molecular markers. The incongruence of phenotype-based classification and molecular phylogeny is a challenge for the classification of these fungi. Additional studies will be needed to determine whether the lineages found here represent cryptic species or whether new phenotypical characters can be found to distinguish these distinct lineages (as has been found in some other Parmeliaceae [10], [57]). Future research should address how such parallel evolution of phenotypical characters in lichenized fungi could be explained in order to provide a better framework to test the adaptive value of these characters [58]. Our results here have important implications for conservation and ecological issues, since species were found to have much more restricted distribution than previously thought.

## Materials and Methods

### Taxon sampling

Eighty two specimens of the *Xanthoparmelia pulla* group from California, the Mediterranean basin, Macaronesia, South America, Australia and South Africa were used for the phylogenetic study. The specimens were identified following the current species delimitations [38], [44], [47], [59], [60]. Chemical constituents were identified using thin layer chromatography (TLC) [61]–[64], and gradient-elution high performance liquid chromatography (HPLC) [65]. The major medullary compounds were classified into four major groups based on their chemical structure: 1) Orcinol depsides: olivetoric, divaricatic, stenosporic and glomelliferic acids. 2) Orcinol depsidones, physodic, alectoronic and glomelliferonic acids. 3) β-Orcinol depsidones: stictic acid (only present in the outgroup) and hypostictic acid. 4) aliphatic acids: constipatic acid. All necessary permits were obtained for the described field studies. Collecting permits in Australia were all obtained by J.A. Elix (ca. 50 permit numbers for each states and over several years) and in Chile by W. Quilhot. For European locations specific permission was not required, since the locations were neither in privately-owned or protected areas. The field studies did not involve endangered or protected species.

### Molecular study

Total DNA was extracted from frozen lobes of thalli crushed with sterile glass pestles, using the DNeasy Plant Mini Kit (Qiagen) following the manufacturer's instructions and modifications of Crespo et al. [66]. The following primers were used: ITS1-LM [67] and ITS2-KL [68] for nuITS rDNA, and LR0R and LR5 [69] for nuLSU rDNA.

For each amplification we used a reaction mixture of 25 μL, containing: 2.5 μL of 10x DNA polymerase buffer (including MgCl_2_ 2mM) (Biotools), 1.25 μL of each primer, 0.75 μL of DNA polymerase (1U/μL), 0.5 μL of dNTPs containing 10 mM of each base (Biotools), 5 μL of DNA (third elution of DNA extraction) and 13.5 μL dH_2_O. Amplifications were carried out in an automatic thermocycler (Techne Progene 3000) with the following steps: an initial denaturation at 94°C for 5 min; 35 cycles of 94°C for 1 min, 58°C (nuITS rDNA) or 56°C (nuLSU rDNA) for 1 min, and 72°C for 1.5 min; a final extension at 72°C for 5 min. PCR products were cleaned with DNA Purification Kit (Flavorgen) and sequenced with the same primers using the ABI Prism Dye Terminator Cycle Sequencing Ready reaction kit (Applied Biosystems) with the following program: initial denaturation at 94°C for 3 min, 25 cycles at 96°C for 10s, 50°C for 5s and 60°C for 4 min. Sequencing reactions were electrophoresed on a 3730 DNA analyzer (Applied Biosystems). The sequence fragments were assembled with Bioedit v. 7.0 [70] and manually adjusted.

### Sequence alignment and selection of the substitution model

We used a dataset of 2 loci of 82 specimens representing 25 species of the *Xanthoparmelia pulla* group and 6 specimens as outgroup. The sequences were mainly generated in this study (140 sequences) and the others taken from our previous studies [33], [41]. The outgroup selection was based on previous phylogenetic studies [41]. GenBank accession numbers and sources of the specimens are listed in Table 2.

The two loci were aligned separately with Muscle 3.6 [71] and the ambiguous positions were removed manually. The general time reversible model including estimation of invariant sites (GTR+I+G) was selected by jModelTest v 0.1.1 [72] as the most appropriate nucleotide substitution model for both loci.

### Phylogenetic Analyses

Potential conflict between the two loci was assessed by comparison of the ML analyses obtained with Garli 0.96 [73] for each locus, using 100 pseudoreplicates for the bootstrap analyses. The phylogenetic analyses of the combined matrix were done using maximum likelihood (ML) and a Bayesian approach. ML analysis was performed using Garli 0.96 [73] with default settings and 100 replicates for the bootstrap analyses. The Bayesian analysis was performed using MrBayes 3.1.1 [74] using the GTR+I+G model, and the data set partitioned into nu ITS and nu LSU. Each partition was allowed to have its own parameter values [75]. Heating of chains was set to 0.2, with 5 million generations sampled every 500th tree. The first 1000 trees were discarded as burn in. We used AWTY [76] to compare splits frequencies in the different runs and to plot cumulative split frequencies to insure that stationarity was reached. Of the remaining 18000 trees (9000 from each of the parallel runs) a majority rule consensus tree with average branch lengths and posterior probabilities was calculated using the sumt option of MrBayes. Clades with bootstrap support equal or above 70 % under ML and/or posterior probabilities ≥0.95 in the Bayesian analysis were considered as strongly supported. Phylogenetic trees were visualized using the program FigTree [77].

### Calibration of nodes and dating analysis

The ages of the *X. pulla* group and its major clades were estimated by a divergence time analysis based on a calibrated phylogeny of the parmelioid lichens [78]. We used a matrix of two loci (nu ITS and LSU) with a proportional number of samples of each parmelioid clade to have a representative tree and trend in speciation through time [79]. The matrix included 299 specimens of parmelioid lichens and 3 specimens of the genus *Usnea* (as outgroup); 62 new sequences of *Xanthoparmelia* species outside the *X. pulla* group were included. GenBank accession numbers with the specimens of the dating analysis are listed in Table S1. The sequence alignment, selection of the nucleotide substitution model, and phylogenetic analyses were done using the same procedures used for the *Xanthoparmelia pulla* dataset (see above).

The divergence time analyses were performed using BEAST v.1.6.1 [80]. We used a starting tree obtained from a ML analysis using Garli 0. 96 [73] of the concatenated dataset, calculated an ultrametric tree using nonparametric rate smoothing (NPRS) implemented in TreeEdit v.10a10 [81]. The age of the crown node of the parmelioid lichens was calibrated at 60 Ma, following Amo de Paz et al. [78]. The starting tree was topologically congruent with the parmelioid phylogeny presented in Crespo et al. [32].

For the divergence time analyses we used two points of calibration: the age of the crown node of the parmelioid lichens set at 60.28 Ma (49.81 – 73.55 Ma) [78], and the age of the crown node of the genus *Parmelia* (dated from the fossil *Parmelia ambra* from the Dominican amber, 15–45 Ma, [82] as discussed previously) [78].

The BEAST analysis was performed using the GTR+I+G substitution model, a Birth-Death process tree prior, and a relaxed clock model (uncorrelated lognormal) for the concatenated dataset. Calibration points were defined as prior distribution, minimal ages and calibrated with a lognormal distributions: 1) the parmelioid crown node at uniform distribution between 49 – 73 Ma; 2) the *Parmelia* crown node at log-normal mean  = 2.77, offset  = 14, lognormal standard deviation  = 0.5. The analysis was run for 10 million generations, with parameter values sampled every 1000 generation. We checked the stationary plateau with Tracer v. 1.5 [83]. We discarded 10% of the initial trees as burn in and the consensus tree was calculated using Tree Annotator v 1.6.1 [80]. The results were visualized with FigTree v. 1.3.1 [84]. Ages of the *X. pulla* clades were estimated for nodes with more than 0.95 of posterior probability in the BEAST runs and in the previous Bayesian analysis.

### Ancestral range reconstructions

The biogeographical analysis to reconstruct the ancestral area was performed using an indirect Bayesian approach to character state reconstruction [85] implemented in SIMMAP v1.5 [86] following [87]. This analysis integrates the combination of the uncertainty in the tree, branch lengths and the substitution models using Markov chain Monte Carlo. We treated the biogeographic regions as discrete characters. The major areas in which *X. pulla* species are distributed were categorised broadly into five areas: California, Mediterranean basin (including Macaronesia), South America, South Africa, and Australia. Presence/absence was coded as binary states and each area was given equal probability. We performed the ancestral state reconstruction analysis on a sub-sample of 1000 trees derived from the MrBayes tree sampling of the *Xanthoparmelia pulla* group.

We also performed ancestral range reconstruction analysis using dispersal-extinction-cladogenesis (DEC) implemented in Lagrange program [88]. The results were inconclusive due to the lack of confidence in parts of the *X. pulla* phylogeny and hence the results are not included in this paper.

## Supporting Information

Figure S1 **Chronogram of parmelioid lichens focusing in** ***Xanthoparmelia pulla*** **group**. Calibration points: A, inferred age of radiation of parmelioid lichens and B, age of the *Parmelia* fossil. The *Xanthoparmelia pulla* group is highlighted by a box and the dated clades are indicated by a branch in bold.
(TIF)

Table S1 **GenBank accession numbers of parmelioid lichens** (**except X. pulla group, see** **Table 2**) **used for divergence time analysis**. New sequences are in bold.
(DOC)
