# Supplementary figures and images for: Transoceanic Dispersal and Subsequent Diversification on Separate Continents Shaped Diversity of the Xanthoparmelia pulla Group (Ascomycota)
Source: PLoS One. 2012 Jun 20;7(6):e39683. doi: 10.1371/journal.pone.0039683 (PMC3379998; doi:10.1371/journal.pone.0039683)

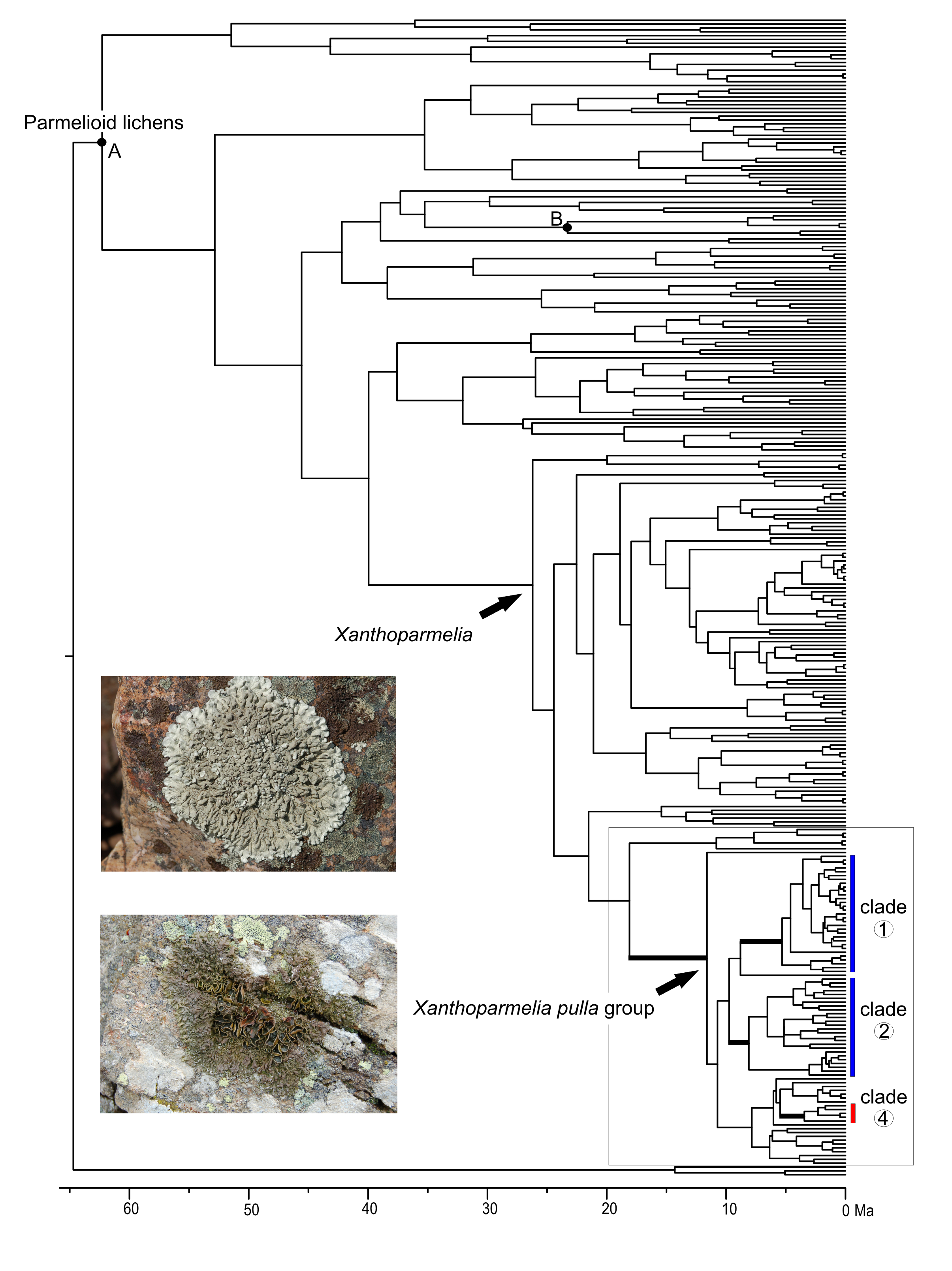

Supplement: Figure S1 — Chronogram of parmelioid lichens focusing in Xanthoparmelia pulla group. Calibration points: A, inferred age of radiation of parmelioid lichens and B, age of the Parmelia fossil. The Xanthoparmelia pulla group is highlighted by a box and the dated clades are indicated by a branch in bold. (TIF) [file pone.0039683.s001.tif]
