# Supplementary material for: Transoceanic Dispersal and Subsequent Diversification on Separate Continents Shaped Diversity of the Xanthoparmelia pulla Group (Ascomycota)
Source: PLoS One. 2012 Jun 20;7(6):e39683. doi: 10.1371/journal.pone.0039683 (PMC3379998; doi:10.1371/journal.pone.0039683)
Supplement: Table S1 — GenBank accession numbers of parmelioid lichens (except X. pulla group, see Table 2 ) used for divergence time analysis. New sequences are in bold. (DOC) [file pone.0039683.s002.doc]

|  | GenBank Acc. No. | |
| --- | --- | --- |
| species | nuITS | nuLSU |
| *Austroparmelina pseudorelicina* | GU994540 | GU994583 |
| *Bulbothrix apophysata* | DQ279481 | EU562670 |
| *B. coronata* | DQ279482 | EU562671 |
| *B. decurtata* | DQ279483 | EU562672 |
| *B. goebelii* | DQ279484 | EU562673 |
| *B. meizospora* | AY611068 | AY607780 |
| *B. setschwanensis* | AY611069 | AY607781 |
| *Canoparmelia crozalsiana* | AY586571 | AY584831 |
| *Cetrariastrum dubitans* | GQ919270 | GQ919246 |
| *Cetrelia chicitae* | AF451759 | DQ923658 |
| *C. olivetorum* | AF451763 | DQ923659 |
| *Everniastrum cirrhatum* | DQ279487 | AY607782 |
| *E. lipidiferum* | DQ279488 | EU562675 |
| *E. nepalense* | DQ383642 | AY607783 |
| *E. rhizodendroideum* | DQ279489 | EU562676 |
| *E. sorocheilum* | DQ279490 | EU562677 |
| *E. vexans* | DQ279491 | EU562678 |
| *Flavoparmelia baltimorensis* | AY586559 | AY584833 |
| *F. caperata* | AY586561 | AY584639 |
| *F. citrinescens* | GU994550 | GU994596 |
| *F. haysomii* | DQ299904 | GU994597 |
| *F. marchantii* | DQ299905 | GU994598 |
| *F. soredians* | AY586562 | AY584835 |
| *F. springtonensis* | EF042907 | EF042916 |
| *F. subambigua* | GU994551 | GU994599 |
| *Flavopunctelia flaventior* | AY773127 | DQ912335 |
| *F. flaventior* | AY773126 | AY578923 |
| *F. soredica* | AY773128 | GU994600 |
| *Hypotrachyna aff taylorensis* | DQ279529 | EU562681 |
| *H. booralensis* | DQ279493 | EU562682 |
| *H. endochlora* | DQ279496 | AY607784 |
| *H. exsecta* | DQ279498 | EU562684 |
| *H. imbricatula* | DQ279503 | EU562686 |
| *H. immaculata* | DQ279504 | AY607785 |
| *H. laevigata* | AY611074 | AY607786 |
| *H. neodissecta* | DQ279510 | EU562689 |
| *H. osseoalba* | DQ279512 | EU562690 |
| *H. physcioides* | DQ279515 | JN939618 |
| *H. pseudosinuosa* | DQ279517 | EU562692 |
| *H. revoluta* | DQ279523 | AY607787 |
| *H. rockii* | DQ279524 | EU562693 |
| *H. sinuosa* | DQ279527 | AY607788 |
| *H. taylorensis* | DQ279528 | AY578924 |
| *M. fuliginosa* | AY611124 | AJ421428 |
| *M. glabra* | AY611114 | AJ421427 |
| *M. subargentifera* | AY611098 | AJ421429 |
| *M. subaurifera* | AY611118 | AJ421432 |
| *Melanohalea aff elegantula* | AY611120 | AY607834 |
| *M. aff exasperata* | AY611092 | AY607804 |
| *M. elegantula* | AY611094 | AJ421437 |
| *M. exasperata* | AY611083 | AJ421438 |
| *M. exasperatula* | AY611090 | AJ421436 |
| *M. olivacea* | AY611091 | AY607803 |
| *M. septentrionalis* | AY611093 | AY607805 |
| *M. subelegantula* | AY611115 | AY607829 |
| *M. subolivacea* | AY611123 | AY607837 |
| *Myelochroa irrugans* | DQ394385 | AY607815 |
| *M. metarevoluta* | AY611102 | AY607814 |
| *Parmelia discordans* | AY583212 | EF042918 |
| *P. saxatilis* | AF058037 | AY578947 |
| *P. saxatilis* | AF141370 | AY300849 |
| *P. serrana* | AF350031 | AY578948 |
| *P. squarrosa* | AY036979 | AY607816 |
| *P. sulcata* | AY580313 | AY578949 |
| *Parmelina carporrhizans* | DQ273854 | DQ268552 |
| *P. carporrhizans* | DQ273853 | DQ268551 |
| *P. coleae* | DQ273856 | DQ268554 |
| *P. coleae* | DQ273855 | DQ268553 |
| *P. pastillifera* | AY611104 | AY607817 |
| *P. quercina* | DQ273848 | DQ268546 |
| *P. tiliacea* | AY581084 | AY578950 |
| *Parmelinella wallichiana* | AY611106 | AY607819 |
| *Parmelinopsis cryptochlora* | DQ279535 | EU562695 |
| *P. horrescens* | DQ279537 | AY578951 |
| *P. minarum* | DQ279539 | AY578952 |
| *P. neodamaziana* | AY611107 | AY607820 |
| *P. subfatiscens* | AY611108 | AY607821 |
| *Parmeliopsis ambigua* | AF451764 | AY607822 |
| *P. hyperopta* | AY611109 | AY607823 |
| *Parmotrema cetratum* | AY586576 | AY584847 |
| *P. crinitum* | AY586565 | AY584837 |
| *P. fistulatum* | AY581057 | AY578920 |
| *P. haitiense* | AY581055 | AY578918 |
| *P. hypoleucinum* | AY586567 | AY584839 |
| *P. perforatum* | AY586568 | AY584840 |
| *P. perlatum* | AY586566 | AY584838 |
| *P. pilosum* | AY581056 | AY578919 |
| *P. reticulatum* | AY586577 | AY584848 |
| *P. robustum* | AY586569 | AY584841 |
| *P. subcaperatum* | AY586557 | AY584829 |
| *P. subthomsonii* | AY586564 | AY584836 |
| *P. subtinctorium* | AY586558 | AY584830 |
| *P. tinctorum* | AB177404 | AY584635 |
| *Pleurosticta acetabulum* | AY581087 | AY578953 |
| *Punctelia borreri* | DQ394373 | AY578954 |
| *P. jeckeri* | AY613407 | GU994625 |
| *P. perreticulata* | AY773124 | GU994626 |
| *P. pseudocoralloidea* | AY586572 | AY584843 |
| *P. reddenda* | AY613410 | GU994627 |
| *P. rudecta* | AY586574 | AY584636 |
| *P. sp.* | GU994579 | GU994628 |
| *P. subflava* | AY586575 | AY584846 |
| *P. subrudecta* | AY773118 | AY578955 |
| *Remototrachyna adducta* | DQ279492 | AY785263 |
| *R. aff brevirhiza* | DQ279494 | - |
| *R. ciliata* | AY785273 | AY785266 |
| *R. costaricensis* | AY785269 | AY785262 |
| *R. crenata* | DQ279495 | EU562683 |
| *R. flexilis* | DQ279500 | EU562685 |
| *R. incognita* | DQ279507 | EU562687 |
| *R. infirma* | DQ279508 | AY785264 |
| *R. koyaensis* | DQ279509 | EU562688 |
| *R. scytophylla* | DQ279525 | EU562694 |
| *Usnea antarctica* | DQ219311 | DQ883692 |
| *U. florida* | AF117996 | EF113545 |
| *U. trachycarpa* | AJ748103 | AJ748103 |
| *Xanthoparmelia aff. epacridea* | HM125752 | HM125777 |
| *X. aff. supposita* | HM125749 | HM125773 |
| *X. aff. supposita* | HM125751 | HM125775 |
| *X. aliphaticella* | **JQ912377** | **JQ912470** |
| *X. aliphaticella* | **JQ912379** | **JQ912472** |
| *X. angustiphylla* | AY581092 | AY578958 |
| *X. atticoides* | AY581066 | AY578929 |
| *X. azaniensis* | EF042900 | EF042910 |
| *X. bibax* | **JQ912373** | **-** |
| *X. brachinaensis* | AY581062 | AY578925 |
| *X. chalybaeizans* | GU903332 | GU903341 |
| *X. competita* | **JQ912383** | **JQ912476** |
| *X. condyloides* | HM125754 | - |
| *X. condyloides* | **JQ912374** | **JQ912468** |
| *X. conspersa* | AY581096 | AY578962 |
| *X. cordillerana* | **JQ912358** | **JQ912453** |
| *X. crespoae* | AY581097 | AY578963 |
| *X. digitiformis* | AY581099 | AY578965 |
| *X. exornata* | EF042908 | EF108318 |
| *X. greytonensis* | HM125755 | HM125780 |
| *X. hottentota* | EF042909 | EF042919 |
| *X. hueana* | AY581090 | AY578956 |
| *X. hypoprotocetrarica* | **JQ912378** | **JQ912471** |
| *X. hypoprotocetrarica* | **JQ912380** | **JQ912473** |
| *X. ianthina* | **JQ912360** | **JQ912455** |
| *X. isidiovagans* | AY581094 | AY578960 |
| *X. lineola* | - | AY578970 |
| *X. lithophila* | AY581077 | AY578941 |
| *X. lithophiloides* | AY581078 | AY578942 |
| *X. mexicana* | **JQ912354** | **JQ912451** |
| *X. mexicana* | **JQ912386** | **JQ912479** |
| *X. murina* | AY581079 | AY578943 |
| *X. neoquintaria* | **JQ912382** | **JQ912475** |
| *X. neotumidosa* | HM125758 | HM125782 |
| *X. norcapnodes* | AY581080 | AY578944 |
| *X. notata* | AY581101 | AY578968 |
| *X. ovealmbornii* | EF042901 | EF042911 |
| *X. peltata* | DQ980021 | DQ923670 |
| *X. perezdepazzii* | **JQ912355** | **-** |
| *X. perspersa* | GU903338 | GU903347 |
| *X. perspersa* | GU992330 | HM125772 |
| *X. perspersa* | GU903339 | GU903348 |
| *X. perspersa* | GU992333 | HM125778 |
| *X. perspersa* | GU992329 | HM125769 |
| *X. perspersa* | HM125758 | HM125783 |
| *X. perspersa* | GU992328 | HM125762 |
| *X. perspersa* | GU992332 | HM125776 |
| *X. protomatrae* | AY581104 | AY578972 |
| *X. protomatrae* | **JQ912376** | **JQ912469** |
| *X. ralla* | GU903337 | GU903346 |
| *X. reptans* | AY581102 | AY578969 |
| *X. saxeti* | AY581063 | AY578926 |
| *X. saxeti* | HM125739 | HM125760 |
| *X. scitula* | GU903333 | GU903342 |
| *X. scitula* | HM125741 | HM125763 |
| *X. scitula* | GU903334 | GU903343 |
| *X. scotophylla* | AY581081 | AY578945 |
| *X. semiviridis* | AY581058 | AY578921 |
| *X. sigillata* | **JQ912359** | **JQ912454** |
| *X. sp.* | **JQ912363** | **JQ912458** |
| *X. sp.* | **JQ912367** | **JQ912462** |
| *X. sp.* | **JQ912357** | **-** |
| *X. sp.* | **JQ912361** | **JQ912456** |
| *X. sp.* | **JQ912366** | **JQ912461** |
| *X. sp.* | **JQ912369** | **JQ912464** |
| *X. sp.* | **JQ912375** | **-** |
| *X. stenophylla* | AY581093 | AY578959 |
| *X. stenophylla* | **JQ912370** | **JQ912465** |
| *X. stenophylla* | **JQ912372** | **JQ912467** |
| *X. subamplexuloides* | HM125753 | HM125779 |
| *X. subchalybaeizans* | **JQ912385** | **JQ912478** |
| *X. subchalybaeizans* | GU903336 | GU993345 |
| *X. subchalybaeizans* | HM125750 | HM125774 |
| *X. subchalybaeizans* | HM125743 | HM125765 |
| *X. subchalybaeizans* | GU903331 | GU903340 |
| *X. subchalybaeizans* | HM125759 | HM125784 |
| *X. subchalybaeizans* | HM125747 | HM125770 |
| *X. subchalybaeizans* | HM125745 | HM125767 |
| *X. subchalybaeizans* | HM125748 | HM125771 |
| *X. subdiffluens* | AY581105 | AY578973 |
| *X. subdiffluens* | **JQ912381** | **JQ912474** |
| *X. subdomokosii* | **JQ912384** | **JQ912477** |
| *X. sublaevis* | AY581106 | AY578974 |
| *X. sublaevis* | **JQ912356** | **JQ912452** |
| *X. subpodochroa* | AY581082 | AY578946 |
| *X. subverrucigera* | AY581091 | AY578957 |
| *X. tentaculina* | HM125756 | HM125781 |
| *X. tinctina* | **JQ912365** | **JQ912460** |
| *X. tinctina* | **JQ912368** | **JQ912463** |
| *X. tinctina* | **JQ912371** | **JQ912466** |
| *X. tinctina* | AY581108 | AY578976 |
| *X. tinctina* | AY581109 | AY578977 |
| *X. tortula* | HM125742 | HM125764 |
| *X. transvaalensis* | AY581095 | AY578961 |
| *X. tzaneenensis* | **JQ912364** | **JQ912459** |
| *X. verrucigera* | **JQ912362** | **JQ912457** |
| *X. verrucigera* | AY581111 | AY578979 |
| *X. vicentei* | AY581112 | AY578980 |

**Table S1. GenBank accession numbers of parmelioid lichens (except X. pulla group, see Table 2) used for divergence time analysis.** New sequences are in bold.
